# Effectiveness and safety of immune checkpoint inhibitors: A retrospective study in Taiwan

**DOI:** 10.1371/journal.pone.0202725

**Published:** 2018-08-24

**Authors:** Jason C. Hsu, Jia-Yu Lin, May-Ying Hsu, Peng-Chan Lin

**Affiliations:** 1 School of Pharmacy and Institute of Clinical Pharmacy and Pharmaceutical Sciences, College of Medicine, National Cheng Kung University, Tainan, Taiwan; 2 Department of Pharmacy, National Cheng Kung University Hospital, Tainan, Taiwan; 3 Department of Internal Medicine, National Cheng Kung University Hospital, Tainan, Taiwan; Roswell Park Cancer Institute, UNITED STATES

## Abstract

**Background:**

Since 2012, several immune checkpoint inhibitors have been approved by the Taiwan FDA for various types of cancer treatment. However, none of them are covered by Taiwan National Health Insurance due to the fact that they are expensive, and there is a lack of clinical evidence as to their effectiveness.

**Objectives:**

This study was aimed toward an exploration of clinical experiences with use of immune checkpoint inhibitors, including indications, prescription types, drug effectiveness, adverse drug event types, and incidence, all of which shall serve as references for future clinical drug use.

**Methods:**

This is a retrospective study focusing on three immune checkpoint inhibitors (ipilimumab, nivolumab, and pembrolizumab), which are available for cancer treatment in Taiwan. We collected data from medical records for the period from January 1st, 2015 to January 12th, 2017 at National Cheng Kung University Hospital (NCKUH), a medical center in southern Taiwan, and recorded these cases until May 31st, 2017. Overall survival (OS) and progression-free survival (PFS) were estimated using the Kaplan-Meier method, and adverse drug reaction odds ratios were analyzed using a chi-square analysis.

**Results:**

The 50 patients under consideration in this study had used any one of the immune checkpoint inhibitors in NCKUH. Non-small cell lung cancer (n = 24, 48%) accounted for the highest percentage, followed by hepatocellular carcinoma (n = 4, 8%). The median OS was not reached, and the PFS for all immunotherapies was 4.9 months. The median OS period and PFS for non-small cell lung cancer (NSCLC) patients were 13 and 4.9 months, respectively, which were similar to those in many clinical trials. For NSCLC patients, the OS and PFS were only 0.63 and 1.37 months for squamous cell type NSCLC, and for patients who were PD-L1 negative, the OS and PFS were only 11.53 and 2.6 months, respectively. The most common adverse events in this study included fatigue (42%), rashes (22%), nausea (20%), and fever (20%), while one patient developed severe deep venous thrombosis and tissue inflammation, which was not confirmed in previous clinical trials.

**Conclusions:**

The histological subtype, the intensity of the PD-L1 expression, and the timing of treatment affected the NSCLC therapeutic results. It is recommended that clinical tests be conducted in order to enhance therapeutic effectiveness. It is expected that more testing, observation-based studies, and research results will validate their efficacy and the tolerance levels of patients.

## Introduction

Immunotherapy is a type of biological therapy that involves either enhancing or inhibiting the immune system to help the body resist foreign diseases, including cancer, infections, or other diseases. Cancer immunotherapy is an issue of considerable concern in academic and clinical fields at present, with a particular emphasis on the development of immune checkpoint inhibitors. The mechanism of immune checkpoint inhibitors is based on PD-1, which acts on T cells. PD-LI or PD-L2 in tumor cells and CD80/86, which inhibits CTLA-4 and antigen-presenting cells, combine to maintain T cell activity, which can be divided into three types: PD-1, PD-L1, and CTLA-4. Among them, PD-1 inhibitors include pembrolizumab and nivolumab; PD-L1 inhibitors include atezolizumab and duravalumab; CTLA-4 inhibitors include ipilimumab and tremelimumab.[1]

The above six drugs have been approved by the US Food and Drug Administration (US FDA), and three of them, including ipilimumab, pembrolizumab, and nivolumab were approved by the Taiwan Food and Drug Administration (TFDA) in 2014, 2015, and 2016 respectively. Ipilimumab was approved to be used for melanoma; pembrolizumab was approved to be used for melanoma and non-small cell lung carcinoma (NSCLC); nivolumab was approved to be used for melanoma, NSCLC, and renal cell carcinoma.[2]

Clinical trial-based research results remain the main sources of immune checkpoint inhibitor information at present. Research on topics including indications, clinical use scenarios, efficacy, and safety regarding the immunotherapies for cancer treatment account for the majority of all literature. In terms of melanoma, compared with chemotherapies, previous studies have found that ipilimumab significantly prolonged patients’ median overall survival (OS) and median progression-free survival (PFS).[3, 4] However, more grade 3 or 4 immune-related adverse events occurred in the ipilimumab group than in the chemotherapy group.[3, 4] Following the use of ipilimumab, pembrolizumab had better PFS and grade 3 or 4 adverse events than was the case with chemotherapies [5], and pembrolizumab also had better objective response rates, OS, PFS, and adverse events in grade 3 and 4 than ipilimumab.[6] Nivolumab’s objective response rate, OS, PFS were also better than chemotherapies, but there was a higher rate of adverse events for grade 3 and 4.[7] Furthermore, several studies compared the objective response rate, OS, PFS, overall adverse events, and adverse events for either grade 3 or 4 between ipilimumab combined with nivolumab and ipilimumab[8–10] or nivolumab[9–11] monotherapy.

As far as NSCLC is concerned, two clinical trials, CheckMate017 and CheckMate057, divided patients into squamous cell and non-squamous cell small-cancer lung cancer groups. KeyNote010 targeted NSCLC patients whose PD-L1 expression exceeded 1%. The previous clinical trials found that pembrolizumab significantly prolonged the OS and PFS of non-squamous cell NSCLC patients, and it had fewer adverse events of grade 3 or 4.[12, 13] Even though some studies have shown that nivolumab has better OS, PFS, and adverse event results as compared to chemotherapies [14, 15], Carbone et al.’s study indicated that its PFS is not better than that of chemotherapies.[16] As for renal cell carcinoma, previous studies have pointed out that nivolumab’s objective response rate, OS, PFS, and adverse events of grade 3 or 4 are better than those of chemotherapies.[17, 18] Previous studies have also compared the efficacy and safety of medication between ipilimumab combined with nivolumab and nivolumab monotherapy and found that the combination’s objective response rate and OS were better than that of monotherapy, but the adverse event rates for the combination were higher than for monotherapy.[19]

Up to the present time, these immune checkpoint inhibitors have not yet been covered by the Taiwan National Health Insurance due to their high costs and inadequate information about their clinical efficacy, including effectiveness, incidence, severity of adverse events, and patient recovery. More observational studies are thus needed in order to make decisions on drug coverage and reimbursement.

The purpose of this study is to gain an insight into National Cheng Kung University Hospital’s (NCKUH’s) clinical experiences with immune checkpoint inhibitor use, including indications, prescription types, drug effectiveness, and adverse drug reaction type and incidence, which will serve as references for future clinical drug use.

## Method

In this study, a record review approach was adopted, and data related to clinical use of immune checkpoint inhibitor drugs were collected from medical records at NCKUH, a medical center in southern Taiwan. The study was conducted in accordance with the protocol approved by the institutional review board (IRB) at National Cheng Kung University Hospital. The IRB waived the requirement for patient informed consent. All of the cancer patients under consideration in this study had used one of the immune checkpoint inhibitors—ipilimumab (50 mg/vial), nivolumab (20 mg/vial and 100 mg/vial), or pembrolizumab (50 mg/vial)—from January 1^st^, 2015 to January 12^th^, 2017. The end of the recorded time was May 31^st^, 2017.

OS, PFS, adverse drug reaction (ADR) type and incidence were recorded until any one of the following termination conditions: (1) any cause of death, (2) reaching the last day of the study period, and (3) lost from observation. In addition, detailed information throughout the patients’ treatment period was collected, including: (1) patient characteristics: medical record number, age, gender, height, weight, body surface area, smoking/alcohol consumption/betel nut chewing, allergy, comorbidity, etc.; (2) long-term medication records (such as medication for hypertension/hyperglycemia/hyperlipidemia, uric acid lowering, pain control, etc.); (3) medical test data: including biochemical, blood, and specific tumor activity indicators for various cancer types, etc.; (4) cancer disease: cancer type, clinical/TNM stages, other treatment methods used prior to the research drug use (prescription and timing), chemotherapy used; (5) treatment and outcomes: prescription pattern of immune checkpoint inhibitors, disease progress, ADR[20], date of death.

Overall survival and progression-free survival (both the mean and median) for patients with immune-therapies for overall cancer and NSCLC treatment were estimated using the Kaplan-Meier method. Patients with NSCLC were also stratified according to gender, age, histological cancer subtype, clinical stage, EGFR mutation, PD-L1, Hepatitis B virus (carriers or not), and timing of treatment (third line or not). We also used the Kaplan-Meier method to compare the OS and PFS among the different groups. Furthermore, the odds ratios of overall ADR and grade 3 or 4 ADR according to previous indicators were estimated using a chi-square analysis.

## Results

### Patient characteristics

Table 1 shows the patient characteristics. From January 1st, 2015 to January 12th, 2017, 50 patients had used any one of the immune checkpoint inhibitors (ipilmumab, nivolumab, pembrolizumab) at NCKUH. The average age of the accepted cases was 58.7 years old (37–80), of which 29 were male, 4 were smokers, and 4 were alcohol consumers. In terms of the laboratory test data, 9 patients were hepatitis B carriers (18%), and 1 patient was hepatitis C antibody positive.

**Table 1:** Cohort’s characteristics.

Characteristics	All patients (N = 50)		Comorbidity	All patients (N = 50)	
	mean	SD		n	%
Age (years)	58.7	11.9	No comorbidities	15	30
Height (cm)	162.5	8.9	Hepatitis B virus carriers	12	24
Weight (kg)	54.7	9.7	Hypertension	10	20
Body surface area (m^2^)	1.6	0.2	Type 2 diabetes mellitus	7	14
SBP (mmHg)	122.1	16.7	Anemia	5	10
DBP (mmHg)	76.6	11.7	Hyperlipidemia	4	8
	n	%	Hyperuricemia	4	8
≧65 years	16	32	Benign prostatic hyperplasia	4	8
Male	29	58	Chronic renal failure	3	6
Smoker	4	8	Peptic ulcer	3	6
Drinker	6	12	Gastroesophageal reflux disease	3	6
Betel nut user	1	2	Hepatitis C	1	2
Drug allergy	13	26	Gout	1	2
Cancer Stage: III	13	26	Liver cirrhosis	1	2
Cancer Stage: IV	37	74	Prior myocardial infarction	1	2
**Laboratory data**	mean	SD	Osteoarthritis	1	2
SCr (mg/dL)	0.8	0.4	Coronary artery spasm	1	2
BUN (mg/dL)	18.2	14.9	Systemic lupus erythematosus	1	2
eGFR (mL/min)	82.3	15.4	Old tuberculosis	1	2
AST (U/L)	57.4	116.1	Generalized anxiety disorder	1	2
ALT (U/L)	28.2	26.1	Pulmonary hypertension	1	2
Direct bilirubin (mg/dL)	0.9	1.5	Chronic obstructive pulmonary disease	1	2
Total bilirubin (mg/dL)	0.9	1.3	Hydronephrosis	1	2
Glucose AC (mg/dL)	108.7	33.4	Hyperthyroidism	1	2
HbA1c (%)	6.7	1.8			
Cholestrerol (mg/dL)	183.9	58.4	**Concomitant medication**	**All patients (N = 50)**	
TG (mg/dL)	152	125.2		n	%
LDL (mg/dL)	125.3	31.7	No concomitant medications	8	16
Na (mmol/L)	137	4.1	Analgesics	20	40
K (mmol/L)	4.1	0.4	Antiviral medication for hepatitis B	10	20
Ca (mmol/L)	9	0.9	Proton pump inhibitors	8	16
P (mmol/L)	3.2	0.7	Oral anti-hyperglycemic drugs	7	14
Mg (mmol/L)	2	0.4	Diuretics	6	12
Hb (%)	11.1	1.5	Steroids	6	12
Hct (%)	33.8	4.2	Calcium channel blockers	6	12
WBC (10^3^/μL)	8.6	5.4	Folic acid	5	10
Plt (10^3^/μL)	239.4	127.3	H_2_ receptor antagonists	8	16
Seg (%)	71.7	11	Anti-hyperuricemic agents	10	20
Eos (%)	2.5	5.1	Alpha blockers	8	16
Lymph (%)	14.6	7.6	Beta blockers	7	14
TSH (μIU/mL)	7.3	26.4	NSAIDs	6	12
TT4 (μg/dL)	7.8	1.1	Aspirin	6	12
TT3 (μg/dL)	95.1	22	Statins	5	10
FT4 (μg/dL)	1.2	0.3	ACEI/ARBs	2	4
CORTI-AM (μg/dL)	28.9	42.3	Ticlopidine	2	4
	n	%	Organic nitrates	1	2
HBsAg (+)	9	18	Clopidogrel	1	2
HCVAb (+)	1	2	Digoxin	1	2
			Sildenafil	1	2
			Warfarin	1	2
			Thyroxine	1	2

Among the patients, 15 patients (30%) were without comorbidity; nearly 25% of the patients were hepatitis B carriers, 10 patients (20%) had hypertension, 7 patients (14%) had diabetes, and 1 person was a hepatitis C patient.

About 15% of the patients were without long-term medication intake; 20 patients (40%) used analgesic drugs, 10 patients (20%) used hepatitis B antiviral drugs, 8 patients (16%) used proton pump inhibitors, and 7 patients (14%) used oral antidiabetic drugs.

### Prescribing pattern

Oncologists prescribed the most immune checkpoint inhibitors (92%), followed by the surgery department (6%), and the chest medicine department (2%). Table 2 shows the immune checkpoint inhibitor indications. Those that meet the indications approved by the Taiwan FDA are mainly NSCLC patients (48%), followed by melanoma (2%), and renal cell carcinoma (2%); of those who meet the indications approved by the US FDA (18%), head and neck cancer patients comprise the majority (16%), as well as ureteral carcinoma (2%); about 30% are off-label use, comprising mainly liver cell carcinoma (8%) and cholangiocarcinoma (6%). All patients that had received immune checkpoint inhibitors were diagnosed with cancer stages 3 or 4.

**Table 2:** Distribution of indications.

Indications			All Patients (N = 50)	
**Taiwan FDA-approved**			**n**	**%**
	Non-small cell lung cancer		24	48
	Melanoma		1	2
	Renal cell carcinoma		1	2
**Off-label use**				
	**US FDA-approved**			
		Tongue cancer	3	6
		Esophageal cancer	2	4
		Hypopharyngeal cancer	2	4
		Nasopharyngeal carcinoma	1	2
		Urothelial carcinoma	1	2
	**US FDA-unapproved**			
		Hepatocellular carcinoma	4	8
		Cholangiocarcinoma	3	6
		Thymic carcinoma	1	2
		Ovarian cancer	1	2
		Lung atypical carcinoid	1	2
		Peritoneal serous adenocarcinoma	1	2
		Prostate cancer	1	2
		Follicular lymphoma	1	2
		Gastric cancer	1	2
		Pancreatic adenocarcinoma	1	2

The prescription types for the immune checkpoint inhibitors used at NCKUH can be generally divided into monotherapy and combination therapy. Combination medical therapy includes chemotherapy, target drugs, radiation therapy, or other immune checkpoint inhibitors. The prescription type is summarized in Table 3.

**Table 3:** Prescribing patterns.

Checkpoint inhibitor		Dosage	Frequency	Other treatments
**Mono-Immunotherapy**				
	Nivolumab	3 mg/kg	Q2W	
	Pembrolizumab	1 mg/kg	Q2W	
	Pembrolizumab	2 mg/kg	Q3W	
	Pembrolizumab	200 mg	Q3W	
	Ipilimumab	3 mg/kg	Q3W	
**Combination immunotherapy**				
	Nivolumab+Ipilimumab	3 mg/kg, 1 mg/kg	Q3W	
	Nivolumab+ Ipilimumab	3 mg/kg, 1 mg/kg	Q3W, Q9W	Bevacizumab+Radiotherapy
**Combination therapy (Immunotherapy+others)**				
	Nivolumab	3 mg/kg	Q2W	Radiotherapy
	Nivolumab	3 mg/kg	Q2W	Sunitinib malate
	Nivolumab	3 mg/kg	Q3W	Bevacizumab
	Nivolumab	3 mg/kg	Q2W	Docetaxel +Cisplatin
	Nivolumab	3 mg/kg	Q2W	Epirubicin+Ifosfamide+ Mesna
	Nivolumab	3 mg/kg	Q3W	Pemetrexed
	Pembrolizumab	2 mg/kg	Q2W	Radiotherapy
	Pembrolizumab	1 mg/kg	Q2W	Gemcitabine
	Pembrolizumab	2 mg/kg	Q3W	Gemcitabine
	Pembrolizumab	200 mg	Q3W	Gemcitabine
	Pembrolizumab	2 mg/kg	Q3W	Cisplatin
	Pembrolizumab	200 mg	Q3W	Cisplatin+ Radiotherapy
	Pembrolizumab	2 mg/kg	Q3W	Pemetrexed
	Pembrolizumab	2 mg/kg	Q3W	Paclitaxel
	Pembrolizumab	200 mg	Q3W	Docetaxel +Cisplatin
	Pembrolizumab	2 mg/kg	Q3W	Docetaxel +Cisplatin
	Pembrolizumab	2 mg/kg	Q3W	Carboplatin+Pemetrexed
	Pembrolizumab	2 mg/kg	Q3W	Cisplatin+Pemetrexed
	Pembrolizumab	2 mg/kg	Q3W	Cisplatin+Fluorouracil+Lecovorin
	Pembrolizumab	200 mg	Q3W	Carboplatin+Fluorouracil

### Drug treatment results

Table 4 shows the treatment effects. Among the 50 patients, 23 (46%) patients died before the end of the study interval; 17 (34%) continued to visit the clinic and could be tracked, and 10 patients (20%) were lost to observation. The median OS was not reached, and the PFS was 4.9 months. Among the NSCLC patients, 2 patients (8%) were diagnosed with squamous cell NSCLC; 4 patients (17%) were stage III; 7 patients (29%) were tested and found to have EGFR mutations; 4 patients (17%) were PD-L1 positive; 4 patients (17%) were Hepatitis B virus carriers, and 6 patients (25%) received the first or second line treatment.

**Table 4:** Effectiveness of immuno-therapies.

Cancer Type				n	Overall Survival (Months)			Progression Free Survival (Months)		
					Mean	Median	Log Rank p-value	Mean	Median	Log Rank p-value
Overall Cancer Types				50	23.37	Didn’t reach		15.00	4.90	
	Non-Small Cell Lung Cancer (NSCLC)			24	11.73	13.00		9.17	4.90	
		Gender	Male	11	11.97	Didn’t reach	0.801	10.13	11.53	0.672
			Female	13	9.70	13.00		7.13	4.43	
		Age	≧65	10	14.07	Didn’t reach	0.175	10.67	5.37	0.416
			<65	14	8.50	11.53		6.27	4.43	
		Histological subtype	squamous cell	2	1.00	0.63	0.010	1.37	1.37	0.272
			non-squamous cell	22	12.70	Didn’t reach		9.47	4.90	
		Stage	IV	20	12.07	Didn’t reach	0.794	9.77	5.37	0.451
			III	4	7.10	2.23		4.57	2.03	
		EGFR mutation	mutation	7	12.13	11.53	0.969	9.90	11.53	0.949
			non-mutation	17	9.67	13.00		7.30	4.90	
		PD-L1	positive	4	13.00	13.00	0.378	10.40	Didn’t reach	0.186
			negative	20	10.80	11.53		8.03	2.60	
		Hepatitis B virus	carriers	4	11.53	11.53	0.453	9.03	11.53	0.559
			non-carriers	20	10.90	13.00		8.50	4.43	
		Timing of treatment	first line treatment	3	9.43	Didn’t reach	0.673	9.43	Didn’t reach	0.446
			second or third line treatment	21	11.50	13.00		8.67	4.90	

During the study period, 11 of the lung cancer patients died; 5 were lost to observation, and 9 reached the last day of the study period. The OS was 13 months, and the PFS was 4.9 months for all NSCLC patients who had used the immune checkpoint drugs. The median OS was not reached, and the PFS for males was 11.53 months, while they the median OS was 13 months, and the PFS was 4.43 months for female patients. For patients aged over 65, the median OS was not reached, and the PFS was 5.37 months, while the median OS was 11.53 months, and the PFS was 4.43 months for patients aged less than 65. Compared with non-squamous cell NSCLC (not reached and 4.9 months), the median OS and PFS for squamous cell NSCLC were much shorter (0.63 and 1.37 months). For patients with EGFR mutation, the OS and PFS were 11.53 months, while they were 13 and 4.9 months for EGFR non-mutation. For patients with PD-L1 positive, the OS and PFS were 13 months and not reached, while they were shorter (11.53 and 2.6 months) for PD-L1 negative patients. For Hepatitis B virus carriers, the OS and PFS were 11.53 months, while they were 13 and 4.43 months for non-carriers. The median OS was 11.53 months, and the PFS was 5.37 months for the third line treatment, while the OS was not reached, and the PFS was 2.43 months for the first line treatment patients.

### Adverse events

#### Laboratory testing

**Serum creatinine** Table 5 shows the adverse events. A total of 45 patients had normal baseline serum creatinine values. Following the drug use, 6 patients (13.33%) (5 used nivolumab, 1 used pembrolizumab) experienced an increase in grade 2 serum creatinine (moderate event), and 1 patient (2.22%) (used pembrolizumab) experienced a grade≧3 event (severe or life-threatening event).

**Table 5:** Safety of immuno-therapies.

			Nivolumab		Pembrolizumab		Ipilimumab		Types of adverse events	All grades		Grade≧3	
			n	%	n	%	n	%		n	%	n	%
**Increasing of Serum Creatinine**									**Fatigue**	21	42	2	4
	baseline		23		20		2		**Rash**	11	22	1	2
	All grades event		5	22	1	5	0	0	**Nausea**	10	20	0	0
		Grade≧3 event	0	0	1	5	0	0	**Fever**	10	20	0	0
**Increasing of AST**									Vomiting	9	18	0	0
	baseline		20		15		0		Anorexia	8	16	1	2
	All grades event		5	25	0	0	0	0	Epigastralgia	5	10	0	0
		Grade≧3 event	1	5	1	7	0	0	Pruritus	5	10	1	2
**Increasing of ALT**									Mucositis	4	8	0	0
	baseline		19		17		1		Diarrhea	3	6	0	0
	All grades event		4	21	3	18	0	0	Numbness	2	4	0	0
		Grade≧3 event	0	0	1	6	0	0	Alopecia	2	4	0	0
**Increasing of Total bilirubin**									Insomnia	2	4	0	0
	baseline		14		10		0		Deep vein thrombosis	1	2	1	2
	All grades event		2	14	3	30	0	0	Cellulitis	1	2	1	2
		Grade≧3 event	1	7	1	10	0	0	Constipation	1	2	0	0
**Hyponatremia**									Arthragia	1	2	0	0
	baseline		14		11		2*		Dry mouth	1	2	0	0
	All grades event		3	21	6	55	0	0	Dysgeusia	1	2	0	0
		Grade≧3 event	1	7	3	27	0	0	Cheilitis	1	2	0	0
**Abnormal serum potassium concentration**													
	baseline		17		15		1*		**Immune-related adverse events**	**All Patients (N = 50)**			
	All grades event		3	18	5	33	0	0		n	%		
		Grade≧3 event	1	6	2	13	0	0	Adrenal insufficiency	2	4		
**Hypoalbuminemia**									Hyperthyroidism	1	2		
	baseline		7		7		0		Hypothyroidism	1	2		
	All grades event		3	43	2	29	0	0	Arthritis	1	2		
		Grade≧3 event	0	0	0	0	0	0	Pneumonitis	2	4		
**Thrombocytopenia**									Hepatitis	1	2		
	baseline		18		10		1						
	All grades event		2	11	2	20	0	0					
		Grade≧3 event	0	0	0	0	0	0					

All grades event = grade 1 (mild) event + grade 2 (moderate) event + grade 3 (severe) event + grade 4 (life-threatening) event

Grade≧3 event = grade 3 (severe) event + grade 4 (life-threatening) event

* Nivolumab + Ipilimumab (n = 1)

**AST value** The base AST test values for a total of 36 patients were normal. After taking medicine, 4 patients (11.11%) (used nivolumab) experienced a grade 1 adverse event (mild event), and 2 patients (5.55%) (1 used nivolumab, and 1 used pembrolizumab) experienced a grade≧3 event.

**ALT value** The base ALT test values for a total of 38 patients were normal. After taking medicines, 7 patients (18.42%) (3 used nivolumab; 1 used nivolumab combined with radiation therapy; 1 used pembroizumab, and 1 used pembrolizumab combined with radiation therapy) experienced a grade 1 adverse event, and 1 patient (2.63%) (used pembrolizumab) experienced a grade≧3 event.

**Total bilirubin** The baseline total bilirubin test values for a total of 24 patients were normal. After taking medicines, 2 patients (20.83%) (used nivolumab or pembrolizumab) experienced grade 1 adverse events; 1 patient (4.17%) (used pembrolizumab) experienced grade 2 events, and 2 patients (8.33%) (used nivolumab or pembrolizumab) experienced a grade≧3 event.

**Blood sodium** The baseline serum sodium test values for a total of 27 patients were normal. After taking medicines, 5 patients (17.86%) (2 patients used nivolumab; 2 used pembrolizumab, and 1 used pembrolizumab combined with radiation therapy) experienced a grade 1 adverse event; 4 patients (8.33%) (1 used pembrolizumab, and 1 used pembrolizumab combined with chemotherapy) experienced a grade≧3 event.

**Serum potassium concentration** The base serum potassium values for a total of 33 patients were normal. After taking medicines, 1 patient (used pembrolizumab) experienced high serum potassium; 4 patients (12.12%) (1 patients used nivolumab; 1 used pembrolizumab, and 1 used pembrolizumab combined with chemotherapy) experienced a grade 1 low serum potassium event; 3 patients (9.09%) (1 used nivolumab; 1 used pembrolizumab combined with radiation therapy, and 1 used pembrolizumab combined with chemotherapy) experienced a grade≧3 low serum potassium event.

**Hypoalbuminemia** The baseline albumin values for a total of 14 patients were normal. After taking medicines, 2 patients (14.29%) (1 used nivolumab, and 1 used pembrolizumab) experienced a grade 1 hypoalbuminemia, and 3 patients (21.43%) (2 used nivolumab, and 1 used pembrolizumab) experienced a grade 2 event.

**Cholesterol and related lipid enzymes** One patient experienced a cholesterol increase following use of ipilimumab and nivolumab. One patient experienced an increase in grade 1 alkaline phosphatase following use of nivolumab, and another patient experienced an increase in grade 1 lipase following the use of nivolumab.

**Nuclear immunity-related test values** Throxine abnormalities occurred in 2 patients after use of nivolumab (one for low thyrotropin, the other hyperthyroidism); low total triodothyronine (TT3) occurred in 4 patients, 2 of which used nivolumab; 1 used nivolumab and ipilimumab combined, and 1 used pembrolizumab combined with chemotherapy. Two patients exhibited excessive cortisol caused by nivolumab and pembrolizumab use.

**Platelets** Thirty patients originally had normal test values for platelets. After taking medicines, 2 patients (6.67%) (who used pembrolizumab and pembrolizumab combined with chemotherapy) had a grade 1 platelet reduction, and 2 patients (6.67%) (who used nivolumab) experienced a grade 2 event.

#### Clinical symptomatic expression of adverse events

Among all the accepted cases, 10 (20%) did not experience adverse drug events. Adverse events can be divided into two categories based on whether or not they are immunity-related. Immunity-related adverse event occurrence types include: adrenal insufficiency (4%), hyperthyroidism (2%) or hypothyroidism (2%), arthritis (2%), pneumonia (2%), and hepatitis (2%); general drug adverse event system occurrence types include: fatigue (2%), rash (22%), nausea (20%), fever (20%), etc. Among them, higher than CTCAE grade≧3 adverse events include: fatigue (4%), rash (2%) urticaria (2%), anorexia (2%), vomiting (2%), deep venous thrombosis (2%), and cellulitis (2%). The results of the chi-square analysis for the odds ratios of overall ADR and grade 3 or 4 ADR by gender, age, histological subtype of cancer, clinical stage, EGFR mutation, PD-L1, Hepatitis B virus and timing of treatment are reported in Table 6.

**Table 6:** Safety of immuno-therapies for NSCLC treatment (n = 24).

Cancer Type			N	Overall ADR						Grade 3 or 4 ADR					
				n	percentage	Odds Ratio	95% C.I. Lower	95% C.I. Upper	Fisher's Exact Test p-value	n	percentage	Odds Ratio	95% C.I. Lower	95% C.I. Upper	Fisher's Exact Test p-value
Non-Small Cell Lung Cancer (NSCLC)			24	19	79%					3	13%				
	Gender	Male	11	7	64%	0.15	0.01	1.58	0.14	2	18%	2.67	0.21	34.20	0.58
		Female	13	12	92%					1	8%				
	Age	≧65	10	7	70%	0.39	0.05	2.92	0.62	2	20%	3.25	0.25	41.91	0.55
		<65	14	12	86%					1	7%				
	Histological subtype	squamous cell	2	1	50%	0.22	0.01	4.36	0.38	1	50%	10.00	0.44	228.70	0.24
		non-squamous cell	22	18	82%					2	9%				
	Stage	IV	20	16	80%	1.33	0.11	16.48	1.00	2	10%	0.33	0.02	4.93	0.44
		III	4	3	75%					1	25%				
	EGFR mutation	mutation	7	6	86%	1.85	0.17	20.26	1.00	1	14%	1.25	0.10	16.50	1.00
		non-mutation	17	13	76%					2	12%				
	PD-L1	positive	4	4	100%	NA	NA	NA	0.54	0	0%	NA	NA	NA	1.00
		negative	20	15	75%					3	15%				
	Hepatitis B virus	carriers	4	4	100%	NA	NA	NA	0.54	0	0%	NA	NA	NA	1.00
		non-carriers	20	15	75%					3	15%				
	Timing of treatment	first line treatment	3	3	100%	NA	NA	NA	1.00	1	33%	4.75	0.29	78.74	0.34
		second or third line treatment	21	16	76%					2	10%				

## Discussion

In previous clinical trials, the median OS of patients using nivolumab or pembrolizumab for first line treatment ranged from 14.4[16] to 22.1[21] months, and for non-first line treatment, ranged from 9.2[15] to 12.7[13] months.[14, 22, 23] One cohort study conducted in France focused on patients with brain metastases. It was found that the median OS was 7.5 months [24], and another study conducted in Spain showed that it was not reached.[25] Based on the results of this study, the median OS for first line treatment was not reached, and it was 13 months for non-first line treatment, which is similar to the findings from clinical trials but it is longer than that in cohort studies. On the other hand, according to the results of previous clinical trials, the median PFS of immune-therapies for first line treatment ranged from 4.2 [16] to 13 [26] months [12, 21, 27], and for non-first line treatment, it ranged from 2.3[14] to 4[13] months.[15, 22] The results of the cohort studies for the median PFS was 2.8 months.[24, 25] Based on the results of this study, the median PFS for first line treatment was not reached and was 4.9 months for non-first line treatment, which was a little bit longer than the findings from clinical trials and cohort studies.

Additionally, clinical trials have also explored the associations between tumor expression indicator PD-L1 and survival, and it has been found that the OS and PFS were significantly longer than chemotherapy users when the patient had more PD-L1 expression.[12, 13, 16] Among the NSCLC patients in this study, PD-L1 expression was positive for 4 cases. Compared with patients with PD-L1 negative expression (11.53 and 2.6 months), the median OS and PFS of the cases with PD-L1 positive expression were 13 months and not reached, respectively. Therefore, it is recommended that future drug users consider clinical trial case acceptance situations and that PD-L1 tests be carried out in order to derive better clinical treatment results.

Immunity-related adverse events noted in instruction leaflets include adrenal insufficiency, hyperthyroidism or hypothyroidism, arthritis, pneumonia, hepatitis, etc. Adrenal insufficiency and thyroid dysfunction occurred in 2 patients in this study (one with hyperthyroidism, the other with hypothyroidism). One of the 2 patients had hyperthyroidism comorbidity, while the other patient’s tumor was a type of large cell neuroendocrine carcinoma (LCNEC). It was also recorded in the study that adrenal insufficiency and thyroid dysfunction occurred at similar times. In addition, one patient with an arthritis comorbidity encountered arthralgia after medication use, combined with joint pain and swelling in the four limbs diagnosed as Rheumatoid arthritis-like arthritis. Thus, it is suggested that caution be taken when handling patients with osteoarthritis.

Common adverse events in this study include fatigue (42%), rashes (22%), nausea (20%), fever (20%), etc. The rates for overall adverse events were less than those reports in the previous clinical trial study, with around 70% overall adverse events. Also, the rates of grade≧3 adverse events for fatigue (4%) and rashes (2%) were much less than those in the previous studies (around 15–30%).[12, 16] The adverse events started from immune checkpoint inhibitor use until the patient condition deteriorated, switched to other chemotherapeutic prescription or treatment, the patient was lost to follow-up, or until the end of the study period. Disease deterioration recording was done mainly to avoid failure to distinguish adverse events arising from subsequent treatment. In addition, according to the records used in this study, grade 3 (severe) deep venous thrombosis and tissue inflammation occurred in one case. Currently, some incidences of adverse events are unknown. Hence, clinical medical staff should keep a close watch.

Clinical trials of immune checkpoint inhibitors often exclude hepatitis B- and hepatitis C-infected patients or carriers. However, currently available information or drug instruction leaflets cannot provide information related to drug safety for hepatitis B and hepatitis-infected patients or carriers. Twelve patients included in this study had been diagnosed as hepatitis B carriers, and one was hepatitis antibody positive. Among them, one patient contracted hepatitis. Later, the patient was suspected of hepatitis B recurrence and resistance to Entecavir. Hence, the original drug was switched to Tenofovir. The results of the comparison of overall ADR and grade 3 or 4 ADR between hepatitis B virus carriers and non-carriers showed no statistically significant variations. The above results should serve as a reference for hepatitis carriers and hepatitis-infected patients in regard to clinical immune checkpoint inhibitor use.

Currently, the indications of ipilimumab, nivolumab, and pembrolizumab approved by the FDA and TFDA are not quite the same. The main indication of ipilimumab is melanoma. The indications of nivolumab and pembrolizumab approved by the FDA not only include metastatic melanoma and metastatic NSCLC, but also include head and neck cancer, urinary epithelial cancer, etc. At present, the indications of these three types of drugs approved by the TFDA are drug melanoma, NSCLC, and renal cell carcinoma, but exclude head and neck cancer.

The indicated doses approved by the US FDA and Taiwan FDA vary. For example, nivolumab and pembrolizumab used to treat non-small cell lung cancer are in fixed doses: 240mg and 200mg. Records from the instruction leaflets from foreign manufacturers show that when using two doses (i.e. pembrolizumab 2 mg/kg Q3W and 200mg Q3W) to treat non-small cell cancer patients, the occurrences of adverse reactions and severe adverse reactions are similar. Comparisons of 2mg/kg Q3W are unavailable.

NCKUH’s prescription guidelines were also examined in this study. In addition to complying with the indications and dosages approved by the FDA and TFDA, the drug must be in use for other cancer types. Further, during the immunotherapy period, radiation theory, chemotherapy, or other target drugs are also conjunctively used. However, due to the scarcity of study cases, it was difficult to carry out a more detailed efficacy comparative analysis with confounding adjustments. The efficacy of and tolerance to this type of drug requires more proof based on future clinical trial evidence or observation test data.

## Conclusion

Using a medical record review, data for three types of immune checkpoint inhibitors approved by the Taiwan FDA were compiled. The use situations at NCKUH mainly provide information pertaining to treated cancer histological subtype, prescription type, adverse event type, ratio, severity, and possible occurrence used for clinicians’ reference. It was also found that when it comes to drug use timing, most of the NSCLC patients from the hospital used the drugs as medication after the second line treatment, which somewhat differed from clinical tests, thus showing a gap between overall efficacy and clinical tests. Moreover, for a small number of cases that underwent test indicator PD-L1, the intensity of the indicator expression affected the therapeutic results. Hence, it is recommended that the timing of clinical drug use be taken into account and that PD-L1 be tested in order to enhance therapeutic efficiency. At present, prescriptions within the hospital often combine different chemotherapy or target drugs. It is expected that more tests and research results or observation-based studies will be conducted to validate efficacy and tolerance related to the use of such drugs.

## Supporting information

S1 Table STROBE statement. (DOC)
